# Clinical Sciences—Leading the Way in Competency-Based Biomedical Education

**DOI:** 10.3390/vetsci7010023

**Published:** 2020-02-17

**Authors:** Mark A. Brown, Ilham Alshiraihi, Kelly Hassell, Shari Lanning

**Affiliations:** 1 Department of Clinical Sciences, Colorado State University, Fort Collins, CO 80523-1052, USA; Shari.Lanning@colostate.edu; 2 Epidemiology Section, Colorado School of Public Health, Fort Collins, CO 80523-1052, USA; 3 Institute for Learning and Teaching, Colorado State University, Fort Collins, CO 80523-1052, USA; 4 Department of Ethnic Studies, Colorado State University, Fort Collins, CO 80523-1052, USA; 5 Cell and Molecular Biology Program, Colorado State University, Fort Collins, CO 80523-1052, USA; ilham.alshiraihi@rams.colostate.edu (I.A.); Kelly.Hassell@rams.colostate.edu (K.H.)

**Keywords:** veterinary education, competency-based education, biomedical education, clinical sciences

## Abstract

For decades, educators in the clinical sciences have been at the forefront of innovations in educational practices related to science and medicine. Ultimately, such innovations are often translated and implemented as best practices across the breadth of biomedical disciplines. Far from novel, competency-based approaches to higher education have been around since the 1960s. These have their origins in student outcomes-based models that focus on the assessment of demonstrated competencies through students’ applications of theory, learned in the classroom, to perform a task and/or resolve a defined issue or problem. Despite its long history of contributing to human medical education and, more recently, veterinary medical education, competency-based instruction is still rare in undergraduate biomedical education. Herein, we discuss the value of clinical education in leading the way toward competency-based, undergraduate biomedical programs.

## 1. Introduction

Competency-based approaches to clinical education evolved from student outcomes-based learning models [1]. These models represented a major advancement beyond the earlier traditional methods to education that were steeped in rote memorization, exams that were correspondingly reflective of that, and lectures where students maintained a largely passive role. At the forefront of student outcomes-based education have always been professional programs, such as medicine, where there are “established standards for competence and performance” associated with specific careers [1,2,3]. Outcomes-based education, in turn, has its foundation in what were called mastery-based learning models, Bloom’s taxonomies for cognitive domains [4], and key developments in mastery learning [1]. Ultimately, outcomes-based learning models were associated with several “shifts in educational priorities” that were “instrumental to the development of competency-based” approaches to higher education [1]. These include (1) “clearly establishing what is meant by competence in each subject area and for each learning task” and (2) “identifying how students can demonstrate such competence through actions and performances” [1]. Collectively, these led to changes in the ways of developing and assessing learning outcomes in professional medical education.

In reflecting upon the recent history of health professions education in the USA, it was almost 120 years ago that a group of reports about the instructional practices for training health professionals, most notably the Flexner Report of 1910 [5], kindled truly novel innovations in medical education for clinicians. Similarly, transformative education reforms were realized for public health and nursing as a result of the Welch-Rose [6] and Goldmark [7] reports, respectively. Together, these reports were the impetus for the institution of grounded science into the curricula of health professions programs. This development ultimately prepared health professionals with the science-based approach to human health associated with the rapid extension of human life expectancy realized over the course of the 1900s. This “first generation of reform” [8] resulted in the modern science-focused health curriculum. A “second generation of reform” in health education programs in the mid-1900s established the use of problem-based learning [8]. These reforms delivered us to the 21st century of health education for professions that demand mastery and competence in an increasingly complicated and technologically complex field of medicine.

We are now in the “third generation of reform” marked by the integration of competency-based clinical education. When it comes to defining competencies for the clinical professions, a definition that is widely accepted in medical education was published in the *Journal of the American Medical Association*: “Competency is the habitual and judicious use of communication, knowledge, technical skills, clinical reasoning, emotions, values, and reflection in daily practice for the benefit of the individual and the community being served” [9]. For clinical training, competency-based practices have a long history of successful implementation in medical specialization programs and residencies. However, the use of competency-based educational practices in four-year programs is a more recent advancement and the institution of competency-based medical admissions has emerged just over the past decade. The new Medical College Admission Test (MCAT), launched in 2015, is more systems based and contextually grounded in a way that “tests higher order cognitive ability, placing greater weight on a student’s ability to demonstrate skills and integrate knowledge across the natural, physical, and social sciences, as opposed to testing factual recall within well-defined disciplines” [10].

This shift is occurring in veterinary educational practices as well. As outlined by the Association of American Veterinary Medical Colleges, “Competency-based veterinary education is an approach modeled after competency-based medical education and is designed to prepare graduates for professional careers by confirming their ability to meet the needs of animals and the expectations of society.” The “approach focuses on outcomes-based and learner-centered education and assessment” [11]. This is addressed in a document published by the Association of American Veterinary Medical Colleges Competency-Based Veterinary Education Working Group in 2019 [11]. The report identifies nine domains of competence, each representing a “group of related abilities necessary for veterinary professionals”: (1) Clinical Reasoning and Decision Making, (2) Individual Animal Care and Management, (3) Animal Population Care and Management, (4) Public Health, (5) Communication, (6) Collaboration, (7) Professionalism and Professional Identity, (8) Financial and Practice Management, and (9) Scholarship [11]. Linked to each domain is a group of competencies which have been deemed “core” for veterinary professionals and which can be “assessed in either the clinical context or in the pre-clinical curriculum” [11].

## 2. Discussion

Although competency-based education is becoming well established in health professions training, competency-based pre-health professions education has yet to be integrated at most institutions. Nevertheless, a competency-based approach to preparing future clinical professionals is certainly in line with a 2009 report published by the Association of American Medical Colleges and the Howard Hughes Medical Institute titled, “Scientific Foundations for Future Physicians” which “urges a shift in premedical student preparation from a narrow list of specific course work to a more flexible curriculum that helps students develop broad scientific competencies” [12]. We know that competency-based admission is the way things are heading in the health professions. It is increasingly being emphasized in medical admissions and is a growing topic of discussion in veterinary admissions. If undergraduate biomedical programs are going to keep pace with best practices in biomedical education and preparation for undergraduates who are interested in careers in the biomedical sciences, undergraduate programs are going to have to be responsive to the increasing emphasis on competency-based education.

The report, “Scientific Foundations for Future Physicians,” which “urges a shift in premedical student preparation from a narrow list of specific course work to a more flexible curriculum that helps students develop broad scientific competencies” [12], is just one of many significant reports underscoring the importance of competency-based education when it comes to the health professions. In 2010, the International Commission on Education of Health Professionals for the 21st century published similar findings in the *Lancet* [8]. Their report is titled, “Health Professionals for a new century: transforming education to strengthen health systems in an interdependent world”. They call for competency-based training to “deliver a renaissance in a new kind of biomedical professionalism: patient-centered, inter-professional, and team-based.” This report highlights what it terms the emergence of a “slow-burning crisis in the mismatch of professional competencies to patient and population priorities because of fragmentary, outdated, and static curricula producing ill-equipped graduates” and it concludes that in order to realize transformative learning in the health professions, there must be a “shift from fact memorization to searching, analysis, and synthesis of information for decision making,” all of which is underscored in competency-based approaches. One particularly acute need in biomedical education highlighted in that report is the “ability for health professionals to manage the explosive increase not only in total volume of information, but also in ease of access to it.” The report emphasizes that “health affects the most pressing global issues of our time including socioeconomic development, national and human security” and it also concludes that “like never before, public prominence of health has generated an environment that is propitious for change” [8]. That 2010 report, in turn, was largely informed by other reports on medical education, including: “Future of Medical Education” by the Association of Faculties of Medicine of Canada, “Tomorrow’s Doctors” by the General Medical Council of the United Kingdom, “Reform in educating physicians” by the Carnegie Foundation, “Revisiting Medical Education at a time of expansion” by the Macy Foundation and “A snapshot of medical student education in the USA and Canada” by the Association of American Medical Colleges. “The focus of these reports is on core competencies beyond the command of knowledge and facts. The competencies to be developed include patient-centered care, interdisciplinary teams, evidence-based practice, continuous quality improvement, use of new informatics, and integration of public health. Research skills are valued, as are competencies in policy, law, management, and leadership.” As a result, it states that “undergraduate education should prepare graduates for lifelong learning. Curriculum reforms should include outcomes-based programs tracked by assessment, capacity to integrate knowledge and experiences, flexible individualization of the learning process to include student-selected components, and development of a culture of critical inquiry” [8].

When it comes to the implementation of competency-based education for the biomedical sciences at the undergraduate level, reports such as “Scientific Foundations for Future Physicians” have started the conversation and the momentum has been building as a result of the work of life science faculty as underscored in reports such as “Vision and Change in Undergraduate Biology Education: A Call to Action” published by the American Association for the Advancement of Science [13]. When it comes to competency-based education, what are we actually observing at the undergraduate level? We are feeling the early tremors and we are just beginning to see the vanguard on the horizon. It is happening at various institutions, but it is not being fully implemented across the curriculum at very many places. Who is in vanguard when it comes to competency-based undergraduate education? The University of Maryland, Purdue University, and Miami all responded collaboratively to the “Scientific Foundations for Future Physicians” report with competency-based initiatives in their undergraduate biology programs funded by the Howard Hughes Medical Institute that has been titled the “National Experiment in Undergraduate Science Education”. This, in turn, has laid the groundwork for other programs such as the new competency-based biomedical sciences curriculum of the University of Texas System. Over the next decade, it is anticipated that most undergraduate biomedical programs will, too, follow the lead of professional medical and veterinary programs in reforms which include the implementation of competency-based practices.

## 3. Conclusions

For decades, the clinical sciences have embraced the practice of competency-based education. This approach has trained our current generation of clinicians and data are currently being generated on the enormously positive outcomes this has achieved at the patient level. The time has come for undergraduate biomedical curricula to align their training with that which is the standard of practice at the professional level. Indeed, the clinical sciences are leading the way in competency-based biomedical education.
